# Reversal Agents: What We Have and What We Can Expect

**DOI:** 10.19102/icrm.2018.090403

**Published:** 2018-04-15

**Authors:** Christian T. Ruff

**Affiliations:** ^1^TIMI Study Group, Cardiovascular Medicine Division, Brigham and Women’s Hospital and Harvard Medical School, Boston, MA, USA

**Keywords:** Anticoagulation, atrial fibrillation, bleeding, reversal agents

## Abstract

Clinical trials in patients with atrial fibrillation have demonstrated that non-vitamin K antagonist oral anticoagulants [novel oral anticoagulants (NOACs)] are markedly safer than warfarin with respect to serious bleeding—especially intracranial hemorrhage, the most feared and devastating complication of anticoagulant therapy. Registries and large retrospective database studies have confirmed these findings. Additionally, patients who do experience bleeding while taking NOACs have similar or better outcomes than do patients on warfarin. However, despite these data, many physicians and patients have been reluctant to embrace NOAC use due to their perception that they are not able to effectively manage patients who present with bleeding, particularly without a specific reversal agent or antidote on-hand. With the approval of the first NOAC-specific reversal agent and with others in late-stage clinical development, it is helpful to review how these agents may fit in the framework of managing NOAC-related bleeding.

## Introduction

For more than half a century, warfarin and other vitamin K antagonists (VKAs) have been the only oral anticoagulants available for clinical use. Although highly effective in preventing stroke and systemic embolism in patients with atrial fibrillation (AF), their use is limited by a narrow therapeutic index that necessitates frequent monitoring and dose adjustments, resulting in substantial risk and inconvenience. Since 2010, however, four novel oral anticoagulants (NOACs or non-VKAs) have been approved that inhibit thrombin (dabigatran) or activated factor X (FXa) (rivaroxaban, apixaban, and edoxaban). These agents have several distinct advantages in comparison with warfarin, including rapid onset and offset of action, absence of an effect of dietary vitamin K intake on their activity, fewer drug interactions, and the ability to be given in fixed doses without routine coagulation monitoring due to their predictable pharmacokinetic and pharmacodynamic effects.^1^ Large phase III trials in patients with AF as well as venous thromboembolism have demonstrated that NOACs have a favorable risk–benefit profile compared with that of warfarin. They are as effective, if not more effective, than warfarin in preventing thromboembolism, and are far safer with respect to serious bleeding, particularly with regard to the risk of intracranial hemorrhage, which is decreased by > 50%.^2,3^ In addition, patients who experience a bleed while taking a NOAC have similar or better outcomes than patients on warfarin.^4,5^ Clinical registries and large retrospective database studies have demonstrated consistent results.^6–10^

Despite these data, physicians and patients have been reluctant to embrace NOACs due to concerns that, without a specific reversal agent on hand, they will not be able to effectively manage patients who have a serious bleed or who require urgent procedures.^11^ With the approval of the first NOAC-specific reversal agent and the late-stage clinical development of several others, it is useful to review the evidence supporting these agents and to consider how these agents should be best incorporated into clinical practice. Prior to discussing the NOAC-specific reversal agents, however, it is important to stress the significance of both preventing bleeding and keeping in mind the general supportive measures that should be routinely employed in order to manage all bleeding events, as most patients who bleed will not require a reversal agent.

## Minimizing the risk of bleeding

Selecting the right dose of NOAC is the most important step to minimize bleeding risks. The prescribing information for all NOACs includes dose reduction criteria to avoid a significant excess in drug exposure (primarily due to impaired renal function). The concomitant administration of antiplatelet drugs and non-steroidal anti-inflammatory drugs should be avoided when possible, as concomitant administration of these medications substantially increases bleeding risk. The consideration of a patient’s renal function is also a critical task that should be performed to determine the timing of NOAC discontinuation prior to a procedure. In general, the FXa inhibitors can be stopped 24 hours to 48 hours prior to the procedure, depending on renal function and bleeding risk associated with the intervention, while a longer duration of interruption is necessary for patients on dabigatran with significant renal dysfunction (dabigatran has 80% renal clearance) who are undergoing an intervention with a high risk of bleeding.^12^

## General supportive measures

Given the short half-lives of these medications, minor bleeds may only require temporary discontinuation of anticoagulation for several doses. More significant bleeds, however, may require additional supportive measures including (1) local management (mechanical/surgical); (2) volume resuscitation; and (3) consideration of red blood cell and platelet transfusion, if appropriate.^12–14^ In cases of overdose in patients who took their last NOAC dose within two hours to four hours prior, oral activated charcoal may attenuate absorption of the drug.^15–18^

Nonspecific hemostatic factors that have been studied as potential NOAC reversal agents include prothrombic complex concentrates (PCCs), activated PCCs, recombinant activated factor VII (rFVIIa), and fresh frozen plasma. PCCs are the preferred nonspecific hemostatic agent for NOAC reversal; they are plasma-derived products that contain three (factors II, IX, and X) or four (factors II, IX, X, and VII) clotting factors, in addition to variable amounts of heparin and the natural coagulation inhibitors protein C and protein S. Animal studies have demonstrated that PPCs have a variable ability to normalize anticoagulation parameters and to prevent or attenuate bleeding seen with NOAC usage.^14,19–25^ The limited data available in humans are restricted to healthy volunteers only. In three small, randomized, placebo-controlled studies involving between 12 and 93 patients, PCC use reversed the anticoagulant effects of rivaroxaban and edoxaban, but not of dabigatran.^15,26–28^ There was a dose-dependent relationship with complete reversal with 50 U/kg and a partial reversal with 25 U/kg.

It is unclear whether normalizing coagulation parameters in healthy volunteers translates to improved outcomes in patients who are actively bleeding. Furthermore, the use of these agents in managing bleeding caused by VKAs or in hemophiliac patients has been associated with an increased risk of thrombotic complications.^29–31^ This risk may be higher when activated factors are used.

## Specific reversal agents

### Idarucizumab

**Figure 1:**
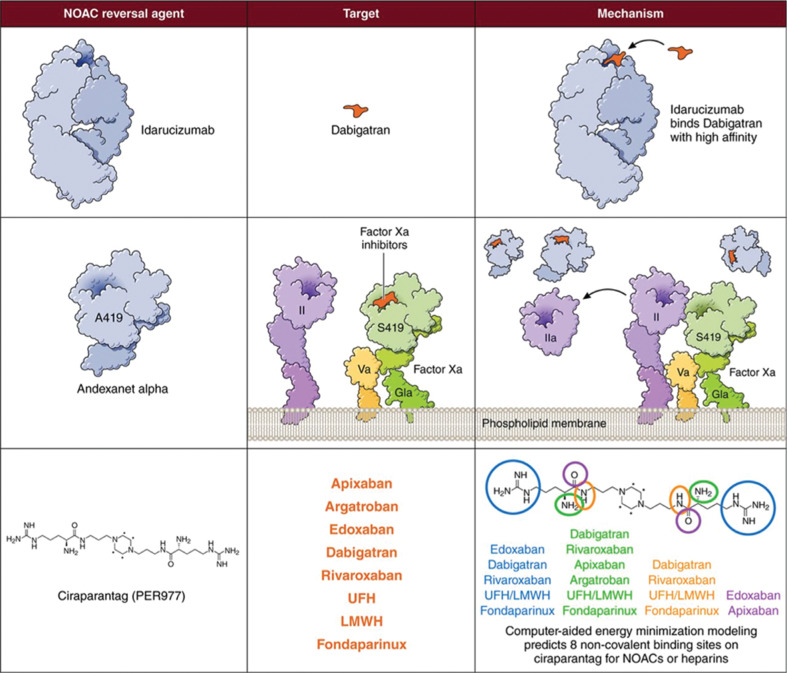
NOAC reversal agents, their targets, and their mechanisms of action. NOAC: novel oral anticoagulant; UFH: unfractionated heparin; LMWH: low-molecular-weight heparin. Reproduced with permission from Ruff CT, Giugliano RP, Antman EM. Management of bleeding with non-vitamin K antagonist oral anticoagulants in the era of specific reversal agents. *Circulation.* 2016;134(3):248–261.

Idarucizumab is a humanized monoclonal antibody fragment developed as a specific reversal agent for dabigatran. It binds with high affinity (350 times higher than that of thrombin) to both free and thrombin-bound dabigatran,^32^ and its binding is effectively irreversible **(Figure 1 and Table 1)**.^33^ In healthy volunteers with normal renal function, peak plasma concentrations were achieved at the end of a five-minute infusion, and idarucizumab was demonstrated to have an initial half-life of 47 minutes.^34^ Despite its short plasma half-life, however, idarucizumab bound to all of the dabigatran present in plasma within minutes.^33^ Idarucizumab is primarily eliminated renally,^34,35^ so drug exposure is increased in patients with impaired renal function. However, such patients also have elevated dabigatran concentrations, since this agent is also predominantly renally cleared.

The Reversal Effects of Idarucizumab on Active Dabigatran (RE-VERSE AD) study (NCT02104947) was a phase III, global, prospective cohort study that investigated the safety and efficacy of 5 g of idarucizumab (administered as two rapid 2.5 g intravenous boluses) in dabigatran-treated patients who presented with uncontrolled or life-threatening bleeding (group A) or non-bleeding patients who required emergent surgery or intervention (group B).^36^ The primary endpoint was the maximum percentage reversal of the anticoagulant effect of dabigatran within four hours of completion of the idarucizumab infusions, on the basis of central laboratory measurement of the patients’ dilute thrombin time or ecarin clotting time. Key secondary endpoints included the time to cessation of bleeding in group A and the assessment of hemostasis during intervention administration in group B. A total of 503 patients (301 in group A and 202 in group B) were enrolled in this study.

Notably, idarucizumab resulted in immediate, complete, and sustained reversal of dabigatran.^36^ The median maximum percentage of reversal of dabigatran was 100% [95% confidence interval (CI): 100–100] as assessed by either dilute thrombin time or ecarin clotting time. Unbound dabigatran concentrations remained below 20 ng/ml (a level that produces little or no anticoagulant activity) for 24 hours in the majority of study subjects; however, a re-elevation of levels above 20 ng/ml occurred in 23% of patients, which was associated with recurrent or continued bleeding in 10 patients (all from group A), with three of these individuals receiving an additional dose of idarucizumab.

In group A, the median time to cessation of bleeding was 2.5 hours. It is important to note that this was investigator-reported (not adjudicated) and that evaluation was only done in 134 out of the 301 patients in this group, as bleeding was not assessed in the 98 patients who presented with intracranial bleeding and could not be determined in 67 additional patients (cases of identified bleeding also stopped prior to treatment in another two patients). In group B, 197 of the 202 patients (97.5%) underwent the intended procedure. The median time from the first infusion of idarucizumab to the initiation of the procedure was 1.6 hours. Periprocedural hemostasis was assessed as normal in 93.4% of the patients, mildly abnormal in 5.1%, and moderately abnormal in 1.5%. Thrombotic events occurred in 24 patients (4.8%) within 30 days and in 34 patients (6.8%) within 90 days, respectively. The 30-day mortality rate was 13.5% in group A and 12.6% in group B, and 18.8% and 18.9%, respectively, at 90 days. There were no serious adverse safety signals.

The United States Food and Drug Administration (FDA) granted accelerated approval to idarucizumab in October 2015, and it is now widely available throughout the world.

### Andexanet alfa

Andexanet alfa (andexanet) is a specific reversal agent for direct (apixaban, rivaroxaban, edoxaban) and indirect (low-molecular-weight heparins, fondaparinux) FXa inhibitors that act through antithrombin **(Figure 1 and Table 1)**. It is a modified human recombinant FXa decoy protein that is catalytically inactive due to the replacement of an active-site serine with alanine and with deletion of the membrane-binding domain, which eliminates the ability to assemble the prothrombinase complex. Andexanet retains the ability to bind to NOACs with high affinity and a 1:1 stoichiometric ratio and, by sequestering FXa inhibitors within the vascular space, endogenous FXa activity is restored.^37^ Because of its pharmacodynamic half-life of one hour, andexanet is administered as a bolus followed by an infusion.

Two parallel, randomized, double-blind placebo-controlled trials [Andexanet Alfa, a Novel Antidote to the Anticoagulation Effects of FXA Inhibitors (ANNEXA) trials] were performed in healthy older volunteers aged 50 years to 75 years who were pretreated with apixaban (ANNEXA-A; NCT02207725) and rivaroxaban (ANNEXA-R; NCT02220725), respectively.^38^ A total of 145 participants were randomized into ANNEXA-A and ANNEXA-R. Based on phase II studies that demonstrated different stoichiometric requirements for different NOACs, a higher dose of andexanet was used for rivaroxaban than for apixaban because of higher plasma concentrations and a larger volume of distribution. For ANNEXA-A with apixaban, andexanet was given as a 400-mg intravenous bolus (30 mg per minute) in part I and as a 400-mg bolus followed by a continuous infusion of 4 mg per minute for 120 minutes (480 mg total) in part II. For ANNEXA-R with rivaroxaban, andexanet was given as an 800-mg bolus in part I and as an 800-mg bolus followed by a continuous infusion of 8 mg per minute for 120 minutes (960 mg total) in part II. The primary endpoint for both studies was the percent change in anti-FXa activity from baseline to nadir. Anti-FXa activity was rapidly (ie, within two to five minutes) reduced by 92% to 94% with andexanet bolus use versus 18% to 21% using the placebo (p < 0.001 for both studies). The reversal of anti-FXa activity persisted for two hours following completion of the bolus administration, although increases were detected within 15 minutes. The reversal was sustained when andexanet was administered as a bolus plus an infusion. Similar decreases were observed for unbound plasma concentrations of apixaban and rivaroxaban. No thrombotic or serious adverse events were reported, and there were no neutralizing antibodies against andexanet or antibodies to factor X or FXa detected. However, transient increases in levels of D-dimer and prothrombin fragments 1 and 2 were observed in a subgroup of participants. The clinical significance of these transient elevations is unknown.

The ongoing ANNEXA-4 (NCT02329327) is a phase IIIb–IV, single-arm, open-label trial evaluating the efficacy and safety of andexanet in patients taking FXa inhibitors with acute major bleeding while receiving the FXa inhibitors apixaban, rivaroxaban, edoxaban, or enoxaparin. Unlike RE-VERSE AD, this study does not include patients who were without bleeding but who required emergency or urgent procedures. In ANNEXA-4, the dosing of andexanet differs depending on the agent used and the timing of the last dose. Patients who have taken apixaban or rivaroxaban more than seven hours prior to the administration of andexanet receive a bolus dose of 400 mg and an infusion dose of 480 mg. For those who have taken enoxaparin, edoxaban, or rivaroxaban at seven hours or less before the administration of andexanet, or for those in whom the timing of the dosage is unknown, these individuals receive a bolus dose of 800 mg and an infusion dose of 960 mg. Coprimary endpoints are the percent change in anti-FXa activity and the rate of excellent or good hemostatic efficacy 12 hours after the andexanet infusion. Hemostatic efficacy in this trial is adjudicated by an independent committee based on predetermined criteria. This is in contrast with RE-VERSE AD, which was investigator-reported.

A preliminary interim analysis of 67 patients demonstrated that an initial bolus reduced anti-FXa activity by 89% (95% CI: 58–94) from baseline among patients receiving rivaroxaban, and by 93% (95% CI: 58–94) from baseline among patients receiving apixaban, with levels that remained similar during the two-hour infusion.^39^ Four hours after the end of the infusion, there was a relative decrease of 39% from baseline in the measure of anti-FXa activity among patients receiving rivaroxaban and a decrease of 30% among those receiving apixaban. Twelve hours after the andexanet infusion, clinical hemostasis was adjudicated as being either excellent or good in 79% of patients (ie, 37 of the 47 patients included in the efficacy analysis). Thrombotic events happened in 12 of 67 patients (18%), and there were 10 deaths that occurred (15% of patients) during the 30-day follow-up period.

Andexanet was initially granted a breakthrough therapy designation by the US FDA, but the agency subsequently requested additional information related to manufacturing and data to support the inclusion of edoxaban and enoxaparin in the label via a Complete Response Letter sent in August 2016. Approval is anticipated in late 2018.

### Ciraparantag

Ciraparantag is a small, synthetic, water-soluble molecule developed as a reversal agent for unfractionated heparin, low-molecular-weight heparins, fondaparinux, and the oral direct FXa and factor IIa inhibitors **(Figure 1 and Table 1)**. It binds to targets through non-covalent hydrogen bonding and charge–charge interactions, thereby preventing the anticoagulants from binding to their endogenous targets.^40^ Ciraparantag was granted a fast-track designation by the FDA, but is at an earlier stage of clinical development in comparison with the other specific reversal agents.

In a phase I dose-ranging study involving healthy volunteers (n = 80) who were administered a single dose of edoxaban 60 mg, ciraparantag decreased whole-blood clotting times to within 10% of baseline values within 10 minutes with single intravenous doses of 100 mg to 300 mg.^41^ Reversal was sustained for 24 hours. Clot formation was restored as assessed by scanning electron micrograph measurement of mean fibrin–fiber diameter. No procoagulant effects or adverse events were reported. An additional study investigating the reinitiation of anticoagulation with edoxaban and a second reversal with ciraparantag is ongoing (NCT02207257).

## Discussion

The availability of NOACs for the prevention of stroke in patients with AF represents a major advance in the treatment of patients. They are as effective, if not more so, than warfarin for the treatment and prevention of thromboembolism, but their real advantage is their improved safety profile, as they cause far less fatal and life-threatening bleeding, particularly intracranial hemorrhage. It is important to emphasize that the enhanced safety of NOACs has been demonstrated repeatedly in clinical trials and patient registries in conjunction with the availability of specific reversal agents or “antidotes,” which underscores that the most important aspect of “managing” bleeding in anticoagulated patients is preventing the bleed from occurring. Unfortunately, however, bleeding will occur with NOAC therapy, and the perceived lack of an ability to reverse the anticoagulant effect is a concern for many patients and physicians and has limited the expansion of anticoagulation into vulnerable patients, such as those who are frail and/or elderly who are deemed to have a prohibitive risk of bleeding.^42^ We now have an opportunity to recalibrate our approach to NOAC therapy and, specifically, the management of bleeding with the availability of the first specific reversal agent idarucizumab for dabigatran, the potential approval of andexanet for the reversal of FXa inhibitors, and the continued development of the universal inhibitor ciraparantag.

The most important impact of the availability of NOAC-specific reversal agents will likely be reassurance, since serious bleeding with NOAC usage is uncommon. The vast majority of bleeds can be managed conservatively with temporary discontinuation of NOACs and supportive measures—time is the only “antidote” required in most cases. Guidelines and institutional protocols will need to be developed to ensure the appropriate use of reversal agents only for patients with serious or life-threatening bleeding, which may include bleeding causing hemodynamic compromise, intracranial hemorrhage, bleeding into a critical organ or closed space, persistent bleeding despite general supportive measures and local hemostatic support, and/or risk of recurrent bleeding due to excess NOAC drug exposure due to delayed clearance of NOAC (eg, acute renal failure) or overdose.^1^ Specific reversal agents are preferred, if available, over non-specific hemostatic agents, as the latter are less effective in reversing coagulation abnormalities, have not been shown to improve outcomes, and are potentially prothrombotic.

While the ability to rapidly and completely reverse the anticoagulant effect of NOACs in a bleeding patient is crucial, an improvement in outcomes may be more limited than expected. Patients with serious bleeding often have an anatomic cause for the bleeding due to a compromise in vascular integrity. Although the presence of an anticoagulant may exacerbate the problem, reversing the anticoagulant effect does not address the primary cause of the bleed. This is reflected in the high 30-day mortality rate of approximately 15% in both RE-VERSE AD^36^ and ANNEXA-4.^39^

## Figures and Tables

**Table 1:** A Comparison of Specific NOAC Reversal Agents

	Idaracizumab		Andexanet Alfa		Ciraparantag	
Chemical structure	•	Humanized monoclonal antibody fragment	•	Recombinant truncated human FXa variant (decoy)	•	Synthetic water-soluble cationic small molecule consisting of two L-arginine units connected with a piperazine-containing linker chain
Binding	•	Noncompetitive binding to dabigatran	•	Competitive binding to direct FXa inhibitors or to indirect FXa inhibitor-activated antithrombin	•	Covalent hydrogen bonding
Target affinity	•	Has an approximately 350-times greater affinity for dabigatran than factor IIa	•	Its affinity for direct FXa inhibitors is similar to that of native FXa	•	Not reported
Onset	•	< 5 minutes	•	2 minutes	•	5–10 minutes
Half-life	•	Initial: 47 minutes	•	Initial: not reported	•	Duration of action: 24 hours
	•	Terminal: 10.3 hours	•	Terminal: ∼6 hours		
Anticoagulant(s) reversed	•	Dabigatran	•	Direct and indirect FXa inhibitors*	•	Dabigatran
					•	Argatroban
					•	Low-molecular-weight heparins
					•	Unfractionated heparin
					•	Oral and parenteral FXa inhibitors
	•	Administered in two doses of 2.5 g intravenously (5 g total) over a period of 5–10 minutes apart				
Route and dose in clinical studies	•	Repeat dosing can be considered if recurrent bleeding occurs or the patient requires a second emergent procedure if elevated coagulation parameters are present	•	Administered as a 400–800 mg intravenous bolus, followed by an infusion of 480–960 mg**	•	Administered as a 100–300 mg intravenous bolus
Storage	•	Requires refrigeration	•	Requires refrigeration	•	At room temperature

NOAC: novel oral anticoagulant; FXa: factor Xa.

*For the indirect FXa inhibitors, andexanet alfa is likely to completely reverse fondaparinux, which only inhibits FXa, but not low-molecular-weight heparins, which also inhibit factor IIa.

**Patients who have taken apixaban or rivaroxaban at more than seven hours prior to administration receive a bolus dose of 400 mg and an infusion dose of 480 mg. For those patients who have taken enoxaparin, edoxaban, or rivaroxaban at seven hours or less before the administration of andexanet, or at an unknown time, they are to receive a bolus dose of 800 mg and an infusion dose of 960 mg.
